# Chitosan as an adjuvant for a *Helicobacter pylori* therapeutic vaccine

**DOI:** 10.3892/mmr.2015.3950

**Published:** 2015-06-17

**Authors:** YANFENG GONG, LIMING TAO, FUCAI WANG, WEI LIU, LEI JING, DONGSHENG LIU, SIJUN HU, YONG XIE, NANJIN ZHOU

**Affiliations:** 1 Departments of Gastroenterology, The First Affiliated Hospital of Nanchang University, Nanchang, Jiangxi 330000, P.R. China; 2 Departments of Obstetrics, The First Affiliated Hospital of Nanchang University, Nanchang, Jiangxi 330000, P.R. China; 3 Department of Biochemistry and Molecular Biology, Jiangxi Medical Science Institute, Nanchang, Jiangxi 330006, P.R. China

**Keywords:** chitosan, *Helicobacter pylori*, vaccine, adjuvant

## Abstract

The aim of the present study was to delineate the therapeutic effect of a *Helicobacter pylori* vaccine with chitosan as an adjuvant, as well as to identify the potential mechanism against *H. pylori* infection when compared with an *H. pylori* vaccine, with cholera toxin (CT) as an adjuvant. Mice were first infected with *H. pylori* and, following the establishment of an effective infection model, were vaccinated using an *H. pylori* protein vaccine with chitosan as an adjuvant. Levels of *H. pylori* colonization, *H. pylori*-specific antibodies and cytokines were determined by enzyme-linked immunosorbent assay. The TLR4 and Foxp3 mRNA and protein levels were determined by reverse transcription polymerase chain reaction and immunohistochemistry, respectively. It was identified that the *H. pylori* elimination rate of the therapeutic vaccine with chitosan as an adjuvant (58.33%) was greater than the therapeutic vaccine with CT as an adjuvant (45.45%). The therapeutic *H. pylori* vaccine with chitosan as an adjuvant induced significantly greater antibody and cytokine levels when compared with the control groups. Notably, the IL-10 and IL-4 levels in the groups with chitosan as an adjuvant to the *H. pylori* vaccine were significantly greater than those in the groups with CT as an adjuvant. The mRNA expression levels of TLR4 and Foxp3 were significantly elevated in the mice that were vaccinated with chitosan as an adjuvant to the *H. pylori* vaccine, particularly in mice where the *H. pylori* infection had been eradicated. The *H. pylori* vaccine with chitosan as an adjuvant effectively increased the *H. pylori* elimination rate, the humoral immune response and the Th1/Th2 cell immune reaction; in addition, the therapeutic *H. pylori* vaccine regulated the Th1 and Th2 response. The significantly increased TLR4 expression and decreased CD4^+^CD25^+^Foxp3^+^Treg cell number contributed to the immune clearance of the *H. pylori* infection. Thus, the present findings demonstrate that in mice the *H. pylori* vaccine with chitosan as an adjuvant exerts an equivalent immunotherapeutic effect on *H. pylori* infection when compared with the *H. pylori* vaccine with CT as an adjuvant.

## Introduction

Epidemiological evidence has indicated a highly significant association between the *Helicobacter pylori* infection and the development of duodenal ulcers and distal gastric adenocarcinoma. In 1994, *H. pylori* was categorized as a class I carcinogen/definite human carcinogen by the World Health Organization (1). Current antibiotic-based therapeutic methods are not practical for global control (2), therefore, vaccines against the *H. pylori* infection are those that were developed in the past (3). *H. pylori* protein vaccines require an effective adjuvant (4) as *H. pylori* proteins exhibit a low immunogenicity, therefore, vaccination with an *H. pylori* antigen alone cannot induce a high enough immune response to deplete the *Helicobacter* infection and protect the gastric mucosa (5). Cholera toxin (CT) and heat-labile *Escherichia coli* enterotoxin (LT) are generally regarded as the most powerful mucosal adjuvants (6,7); however, their use in humans is hampered by their particularly high toxicities. CT and LT have been restructured to reduce their toxicities (8), however this resulted in a reduction of their adjuvant effects.

Chitosan, a polymer of D-glucosamine and a natural product derived from chitin, is accessible, and demonstrates good bioadhesion, biodegradability and biocompatibility without immunogenicity, toxicity or side-effects (9); thus, chitosan has been used in mucosal vaccines as an adjuvant (10).

Numerous studies have indicated that chitosan effectively elicits a local (particularly mucosal local) immune response, enhances the ability of antigenic delivery systems and performs adjuvant activity in vaccines (11). It has been reported that *Neisseria meningitidis* and *Bordetella pertussis* vaccines with chitosan as the adjuvant successfully induced a protective immune response (12).

Our previous study demonstrated that oral administration of *H. pylori* whole-cell sonicate plus chitosan as the adjuvant protected mice against *H. pylori* infection (13). Furthermore, it has been shown that, as an adjuvant in vaccines for *H. pylori* protection, chitosan is more effective than CT in immune protection against *H. pylori* infection (14). However, to the best of our knowledge, there have been no reports regarding chitosan as an adjuvant for the *H. pylori* therapeutic vaccine and the immunoprotection mechanism remains unclear. Therefore, in the present study, mice were infected with *H. pylori* and then vaccinated using an *H. pylori* protein vaccine with chitosan as the adjuvant. This was to delineate the therapeutic effect of the *H. pylori* vaccine and the potential mechanism against *H. pylori* infection in comparison to a *H. pylori* vaccine with CT as the adjuvant.

## Materials and methods

### Reagents and bacterial strains

Chitosan and 88.5% deacetylated chitosan powder were purchased from Shanghai Qisheng Biological Preparation Co., Ltd. (Shanghai, China). Rabbit anti-rat IgG1 (cat. no. PA1-86329; Zymed Life Technologies, Carlsbad, CA, USA), IgG2a (cat. no. 61-0220; Zymed Life Technologies) and IgA (cat. no. Sab3700520; Sigma-Aldrich, St. Louis, MO, USA), and goat anti-mouse IgG (cat. no. A27025; Zymed Life Technologies) peroxidase conjugate were purchased from Zymed Life Technologies (Carlsbad, CA, USA). CT was purchased from Sigma-Aldrich. Enzyme-linked immunosorbent assay (ELISA) kits for interleukin (IL)-2, interferons (IFNs), IL-12, IL-4, and IL-10 were purchased from eBioscience, Inc. (San Diego, CA, USA). Polymerase chain reaction (PCR) primers were purchased from Shanghai Sheng Gong Biological Engineering Technology Service Co., Ltd. (Shanghai, China) Goat anti-mouse TLR4 polyclonal antibody (cat. no. sc-12511) was purchased from Santa Cruz Biotechnology, Inc. (Dallas, TX, USA). Rabbit anti-rat Foxp3 polyclonal antibody (cat. no. bs-10211R) was purchased from Beijing Bo Orson Biological Technology Co., Ltd., (Beijing, China) and the *H. pylori* Sydney strain 1 (SS1) was provided by the *H. pylori* Strain Pool (Chinese Centre for Disease Control, China). An 450 enzyme microplate reader was purchased from Bio-Rad Laboratories, Inc. (Hercules, CA, USA). A PCR thermal cycler was purchased from PerkinElmer, Inc. (Waltham, MA, USA). A JS680C gel imaging analysis system was purchased from Shanghai Peiqing Science and Technology Co., Ltd (Shanghai, China) and the ECP3000 electrophoresis apparatus was purchased from Beijing Liuyi Instrument Factory (Beijing, China). A BH-2 stereo-binocular microscope was purchased from Suzhou REIT Image Technology Co., Ltd. (China).

### Animals

Female BALB/c mice (age, 6–8 weeks; mean weight, 22.5 g) were purchased from the Animal Center of the Chinese Academy of Sciences (Shanghai, China). The mice were housed in a specific pathogen-free environment with free access to food and water. All animal experiments were conducted in accordance with principles stated in the Guide for the Care and Use of Laboratory Animals. The experimental protocols were approved by the Ethics Committee of The First Affiliated Hospital of Nanchang University.

### H. pylori culture

The *H. pylori* SS1 was used throughout the investigation. *H. pylori* was grown in a *Campylobacter* agar base, containing 10% sheep blood, under microaerobic conditions (5% O_2_, 10% CO_2_ and 85% N_2_) at 37°C for 2–3 days.

### Preparation of H. pylori antigen

After culturing for 2–3 days, the *H. pylori* SS1 was eluted with phosphate-buffered saline (PBS) and centrifuged at 10,000 × g and 4°C for 10 min. The pellet was washed and sonicated. Following centrifugation at 8,000 × g and 4°C for 30 min, the supernatant was collected and stored at −80°C until use. The protein concentration was determined using a bicinchoninic acid (BCA) assay.

### Preparation of chitosan particles and solution

Deacetylated (88.5%) chitosan powder was suspended in saline to a final concentration of 10 mg/ml and sonicated twice (output, 80 Hz). The small particles in the supernatant were removed. The chitosan particles were collected by further centrifugation at 140 × g for 10 min. Chitosan stock solution [3% (w/w)] was prepared from 88.5% deacetylated chitosan powder in 0.8% (v/v) acetic acid and 0.9% (w/v) saline.

### H. pylori infection

Each mouse was orally administered with 1×10^9^ colony-forming units (CFUs) of *H. pylori* per liter five times every other day. Twelve weeks after the last inoculation, four mice were euthanized, and the stomachs were removed to ascertain whether the *H. pylori* infection model had been established.

### H. pylori vaccination

The infected BALB/c mice were orally immunized in the following groups at days 0, 7, 14 and 21: i) Control (PBS alone), 12 mice; ii) *H. pylori* antigen alone, 11 mice; iii) *H. pylori* antigen plus 0.5% chitosan solution, 12 mice; iv) *H. pylori* antigen plus CT (5 *µ*g/mouse), 11 mice (one mouse died); and v) *H. pylori* antigen plus chitosan particles (500 *µ*g/mouse), 12 mice. Four weeks after the final vaccination, saliva and blood samples were collected. The stomachs were isolated and cut longitudinally into two sections. One was used for examination of *H. pylori*, and the other was used for histology and immunological assays.

### Assessment of bacterial load in the stomach

The bacterial load in the stomach was determined by quantitative culture of *H. pylori* and Giemsa staining. *H. pylori*-negative was defined when the culture of *H. pylori* and the Giemsa staining were negative. *H. pylori*-positive was defined when either the culture of *H. pylori* or the Giemsa staining was positive. For assessment of *H. pylori* colonization, the weighed stomachs were homogenized in Brucella broth and spread over a serum plate. The plates were incubated for 3–7 days, and the *H. pylori* colonies were counted and calculated as CFUs per gram of stomach tissue. For Giemsa staining, the colonization was assessed by semi-quantitative analysis of *H. pylori* in the gastric mucosa (0, nil; 1, 1–2 cells/crypt; 2, 3–10 cells/crypt; 3, 11–20 cells/crypt; and 4, >21 cells/crypt).

### Determination of H. pylori-specific antibody levels in the gastric mucosa and saliva

The *H. pylori*-specific antibodies, IgG, IgG1, and IgG2a in sera, and IgA in the gastric mucosa and saliva were detected by indirect ELISA. After weighing, the gastric mucosa was homogenized in PBS and the homogenates were centrifuged at 3,000 × g at 4°C for 20 min. The supernatant was harvested and diluted at 1:2. The sera and saliva were diluted at 1:100 and 1:5, respectively. Peroxidase-conjugated rabbit anti-rat IgG1, IgG2a or IgA was diluted at 1:1,000 and peroxidase-conjugated goat anti-mouse IgG secondary antibody was diluted at 1:2,000. The antibody levels of each immunized group from the sera and saliva were represented as relative levels to the mock-immunized control group. The IgA levels in the gastric mucosa were represented as relative levels (per gram wet weight of the gastric mucosa) to the mock-immunized group.

### Determination of cytokines in the gastric mucosa by ELISA

After weighing, the gastric mucosa was homogenized in PBS and the homogenates were centrifuged at 3,000 × g at 4°C for 20 min. ELISA kits were used to quantify IL-2, IFN, IL-12, IL-4 and IL-10 in the supernatants (diluted at 1:2) following centrifugation. The results were represented as pg/mg wet weight of the gastric mucosa.

### Determination of TLR4 and Foxp3 mRNA contents in the gastric mucosa by reverse transcription (RT)-PCR

Total RNA was isolated from the mouse gastric mucosa to determine TLR4 and Foxp3 mRNA levels within the gastric mucosa using RT-PCR. The cDNA from each sample served as a template for subsequent PCR assays to assess the TLR4 and Foxp3 mRNA levels, which were normalized to the expression of β-actin. Each 50-*µ*l PCR consisted of 25 pmol of each primer, 10 mM Tris (pH 8.3), 1.5 mM MgCl_2_, 200 *µ*M dNTPs, and 0.5 *µ*l of Taq enzyme. β-actin and TLR4 were amplified at 95°C for 5 min (1 cycle); 95°C for 30 sec, 56°C for 30 sec and 72°C for 1 min (30 cycles); and 72°C for 5 min (1 cycle). Foxp3 was amplified at 95°C for 2.5 min (1 cycle); 95°C for 30 sec, 57°C for 30 sec and 72°C for 1 min (32 cycles); 72°C for 5 min (1 cycle). The PCR products were visualized following electrophoresis on 2% agarose gels and the band intensities were quantified by densitometry.

### Determination of TLR4 and Foxp3 protein expression in the gastric mucosa by immunohistochemistry

Snap-frozen biopsies were cut into 4-*µ*m sections to determine the levels of TLR4 and Foxp3 protein expression in the gastric mucosa by immunohistochemistry. In the TLR4-stained sections, positive cells were assigned to the cell membrane or to yellow/brown-dyed plasma, and negative cells were assigned to the cell membrane or to non-dyed plasma. In Foxp3-stained sections, positive cells were assigned to the cell nucleus or to yellow/brown-dyed plasma and negative cells were assigned to the cell membrane or to non-dyed plasma. The immunoreaction was graded according to the depth of the color and the proportion of positive cells. The degree of dyeing was divided into the following score grades and expressed as a percentage: 0, Negative; 1, (yellow) weakly positive; 2 (light brown) positive; and 3 (brown) strongly positive.

### Statistical analysis

Differences in the eradication rate were analyzed by Fisher's exact test. Differences in *H. pylori*-specific antibody levels in the gastric mucosa among the experimental groups were evaluated for statistical significance by analysis of variance or Student's t-test. P<0.05 was considered to indicate a statistically significant difference.

## Results

### Animal model of H. pylori infection

**Figure 1:**
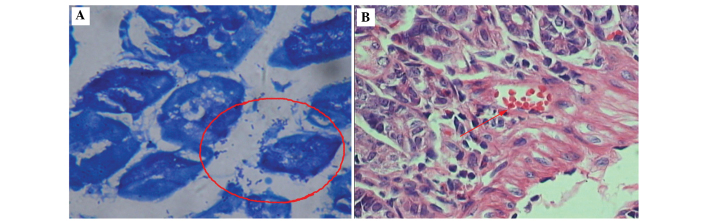
(A) Giemsa staining of the gastric mucosa (magnification, x400). Mice were euthanized 12 weeks after the *H. pylori* inoculation and the tissue sections were stained by Giemsa staining. The red circle indicates *H. pylori* colonization in the gastric pits. (B) Hematoxylin and eosin staining of the gastric mucosa (magnification, x400). Mice were euthanized 12 weeks after the *H. pylori* inoculation. The red arrow indicates mucosal hyperemia, lymphocytes and neutrophil infiltration.

The animal model of *H. pylori* infection was established for the different infection groups. Four mice were euthanized 12 weeks after oral infection with *H. pylori*. Positive *H. pylori*-infection was observed by *H. pylori* culture and Giemsa staining of sections of the infected stomachs (Fig. 1A). Gastric inflammation was observed by hematoxylin and eosin (H&E) staining (Fig. 1B), thus indicating the *H. pylori* infection.

### H. pylori infection among different groups

**Figure 2:**
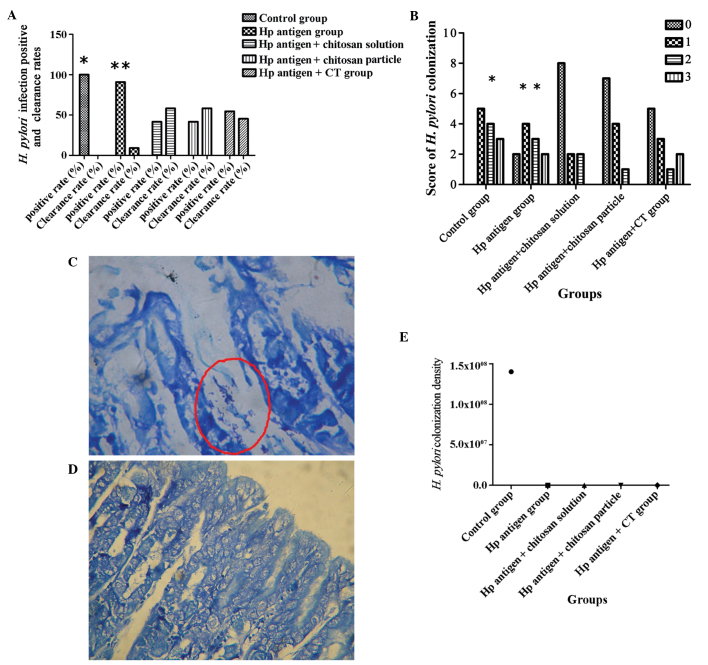
(A) Hp infection in the gastric mucosa after therapeutic vaccination. ^*^P<0.005 compared with the Hp antigen + chitosan solution group and the Hp antigen + chitosan particle group; P<0.05 for the control group vs. the Hp antigen + CT group. ^**^P<0.05 compared with the Hp antigen + chitosan solution group and the Hp antigen + chitosan particle group. (B) The Hp colonization density in the groups with the adjuvants was significantly less when compared with the control group (P<0.05), and the Hp colonization density in the group with chitosan as the adjuvant was significantly less than that of the Hp antigen group without any adjuvant (P<0.05). ^*^P<0.001 compared with the Hp antigen + chitosan solution group and the Hp antigen + chitosan particle group; P<0.05 for the control group vs. the Hp antigen + CT group. ^**^P<0.05 compared with the Hp antigen + chitosan solution group and the Hp antigen + chitosan particle group. (C and D) Giemsa staining of the gastric mucosa (magnification, x400). (0, nil; 1, 1–2 cells/crypt; 2, 3–10 cells/crypt; 3, 11–20 cells/crypt; and 4, >21 cells/crypt). (C) The control group revealed greater Hp colonization in the gastric pits as indicated by the red circle. (D) The group with chitosan as the adjuvant demonstrated no Hp colonization in the gastric pits. (E) The Hp colonization density in the group with chitosan as the adjuvant was significantly less than that of the Hp antigen group without any adjuvants (P<0.05). Hp, *H. pylori*; CT, cholera toxin.

To determine *H. pylori* infection in the gastric mucosa following the therapeutic vaccination *H. pylori* clearance was measured, and the *H. pylori* colonization scores and density of the quantitative *H. pylori* culture were obtained. Pathological tests of the gastric mucosa were also conducted (Fig. 2).

After the therapeutic vaccination, the gastric mucosae of *H. pylori*-infected mice were analyzed for *H. pylori* infection. The *H. pylori* clearance in the groups with chitosan as the adjuvant was identified to be significantly greater than that of the mock-immunized control group (P<0.005) and the *H. pylori* antigen-immunized group without any adjuvant (P<0.05); in addition, the *H. pylori* clearance in the group with CT as the adjuvant was significantly greater than that in the control group (Fig. 2A; P<0.05). The *H. pylori* colonization density in the control group was significantly greater than that in any of the other groups (Fig. 2B and Table I; P<0.05), and the *H. pylori* colonization density in the groups with chitosan as the adjuvant was significantly less than that of the *H. pylori* antigen group without any adjuvants (Fig. 2E and Table I; P<0.05). No significant difference was identified between the groups with chitosan as the adjuvant and the group with CT as the adjuvant (Fig. 2A, B and E; P>0.05).

### Grades of gastritis

*H. pylori* clearance, *H. pylori* colonization scores, *H. pylori* colonization density of the quantitative culture, and grades of acute and chronic gastritis in the gastric mucosa were measured to determine *H. pylori* infection and gastritis in the gastric mucosa following therapeutic vaccination.

**Figure 3:**
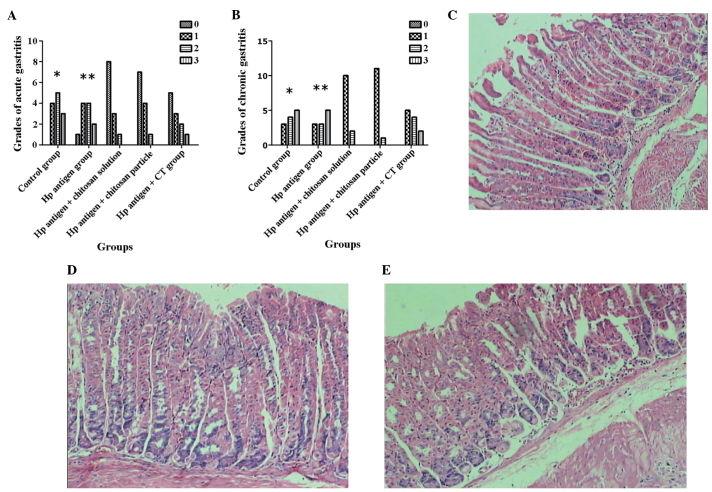
(A) Grade values of acute gastritis. ^*^P<0.001 compared with the Hp antigen + chitosan solution group and the Hp antigen + chitosan particle group; P<0.005 for the control group vs. the Hp antigen + CT group. ^**^P<0.005 compared with the Hp antigen + chitosan solution group and the Hp antigen + chitosan particle group; P<0.05 for the Hp antigen group vs. the Hp antigen + CT group. (B) The grade values of chronic gastritis in the groups with chitosan as the adjuvant were significantly less when compared with those of the groups without chitosan as the adjuvant (P<0.001–0.05). ^*^P<0.001 compared with the Hp antigen + chitosan solution group and the Hp antigen + chitosan particle group. ^**^P<0.05 compared with the Hp antigen + chitosan solution group and the Hp antigen + chitosan particle group. (0, few lymphocyte; 1, sporadic lymphocyte and plasmocyte; 3, more lymphocyte and plasmocyte; 3, large number of lymphocyte and plasmocyte). (C-E) H&E staining of the gastric mucosa (magnification, x100) revealed the following: (C) The control group demonstrated increased inflammatory cell infiltration; (D) the group with chitosan as the adjuvant revealed mild inflammatory cell infiltration; and (E) the group with CT as the adjuvant demonstrated increased inflammatory cell infiltration. Hp, *H. pylori*; CT, cholera toxin; H&E, hematoxylin and eosin.

The gastric mucosae of *H. pylori*-infected mice were tested for gastritis after therapeutic vaccination. The grade of acute gastritis in the groups with an adjuvant was identified to be significantly lower than that of the control group and the *H. pylori* antigen group without any adjuvant (Fig. 3A; P<0.05). For acute gastritis after *H. pylori* infection, no difference was observed in the therapeutic effect between the groups with chitosan as the adjuvant and the group with CT as the adjuvant (Fig. 3A; P>0.05). However, the vaccine with chitosan as the adjuvant relieved chronic gastritis to a greater extent than the vaccine with CT as the adjuvant (Fig. 3B; P<0.05). HE staining of the gastric mucosa of the control group revealed an increased chronic inflammation response (Fig. 3C). The group with chitosan treatment revealed milder chronic inflammation response compared with the group with CT as adjuvant (Fig. 3D and E).

### Levels of H. pylori-specific antibodies in the gastric mucosa serum, and saliva

To determine the local immune response induced by the therapeutic vaccination, the levels of *H. pylori*-specific IgA and IgG antibodies in each group were measured by ELISA.

**Figure 4:**
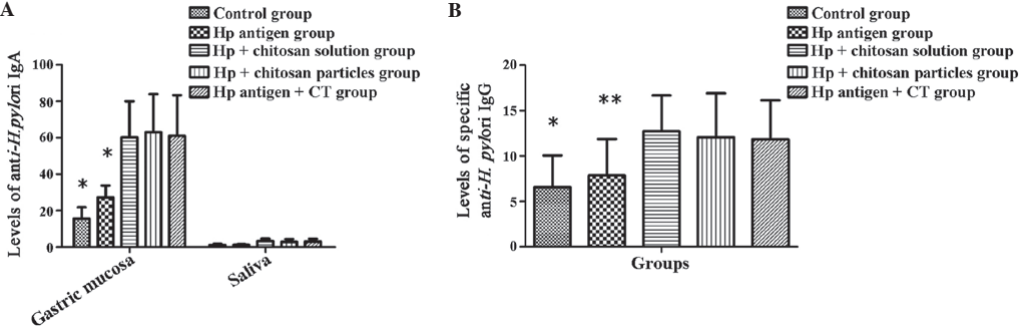
(A) Levels of Hp-specific antibodies in the gastric mucosa and saliva. (B) Levels of Hp-specific antibodies in the sera. ^*^P<0.01 and ^**^P<0.05, compared with the Hp + chitosan solution group, the Hp + chitosan particle group and the Hp antigen + CT group; .Hp, *H. pylori*; CT, cholera toxin.

A significant difference (P<0.001) was observed between the different groups in the level of anti-*H. pylori* IgA in the gastric mucosa and saliva, and specific anti-*H. pylori* IgG in the sera. The levels of *H. pylori*-specific antibodies in the groups that were treated with a vaccine plus an adjuvant were significantly greater when compared with those of the control group and the *H. pylori* antigen groups (Fig. 4A and B; P<0.01-0.001). In addition, no significant difference was identified between the groups with chitosan as the adjuvant and the group with CT as the adjuvant (Fig. 4A and B; P>0.05).

### Effect of TH1 and TH2

To determine the cellular immune response (TH1 and TH2) induced by the therapeutic vaccination, ELISA was conducted to establish the levels of cytokines in the gastric mucosa and the ratio of IgG2a to IgG1 in the sera of each group.

**Figure 5:**
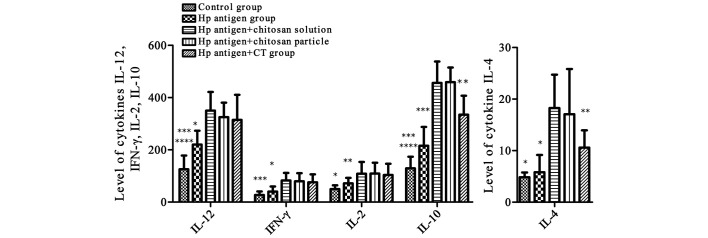
Levels of Hp-specific antibodies in the gastric mucosa, serum and saliva. ^*^P<0.01, ^**^P<0.05 and ^***^P<0.001 between the Hp antigen group and the Hp + chitosan solution group, the Hp + chitosan particle group, and the Hp antigen + CT group. Hp, *H. pylori*; CT, cholera toxin.

The levels of cytokines, IL-12, IFN-γ, and IL-2 in the groups administered with a vaccine plus an adjuvant were observed to be significantly greater than those of the control group and the *H. pylori* antigen group (P<0.001–0.05). The level of IL-10 in the groups administered with a vaccine plus an adjuvant was significantly greater than that of the control and the *H. pylori* antigen groups (P<0.001–0.01), and the level of IL-10 in the group administered with a vaccine plus chitosan as the adjuvant was identified as significantly greater than that of the group with CT as the adjuvant (P<0.01). The level of IL-4 in the group administered with a vaccine plus chitosan as the adjuvant was significantly greater than that of the control group, the *H. pylori* antigen group and the group with CT as the adjuvant (P<0.001–0.05). The level of IL-10 in the group administered with a vaccine plus CT as the adjuvant was found to be significantly greater than that of the control group (Fig. 5; P<0.05).

**Figure 6:**
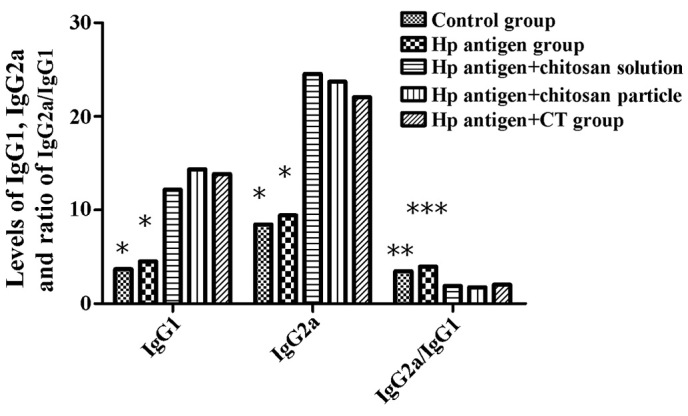
Levels of IgG1 and IgG2a, and ratio of IgG2a to IgG1 in the sera. ^*^P<0.001 vs. the Hp antigen + chitosan solution group, the Hp antigen + chi-tosan particle group, and the Hp antigen + CT group. ^**^P<0.05 vs. the Hp antigen + chitosan solution group and the Hp antigen + chitosan particle group. ^***^P<0.01 vs. the Hp antigen + chitosan particle group, ^***^P<0.05 when compared with the Hp antigen + chitosan solution group and the Hp antigen + CT group. Hp, *H. pylori*; CT, cholera toxin.

The ratio of IgG2a to IgG1 in the sera of the groups administered with a vaccine plus an adjuvant was significantly less than that of the control and the *H. pylori* antigen groups (Fig. 6; P<0.01–0.05).

### TLR4 mRNA and protein expression levels in the gastric mucosa

To determine the role of TLR4 in vaccination therapy following *H. pylori* infection, TLR4 mRNA and protein expression levels in the gastric mucosa were measured by RT-PCR and immunohistochemical staining. The primer to amplify the 348-bp β-actin cDNA were as follows: Forward, GAC GAT ATC GCT GCG CTG and reverse, GTA CGA CCA GAG GCA TAC AGG. The primer to amplify the 438-bp TLR4 cDNA were as follows: Forward, CAG CTT CAA TGG TGC CAT CA and reverse, CTG CAA TCA AGA GTG CTG AG.

**Figure 7:**
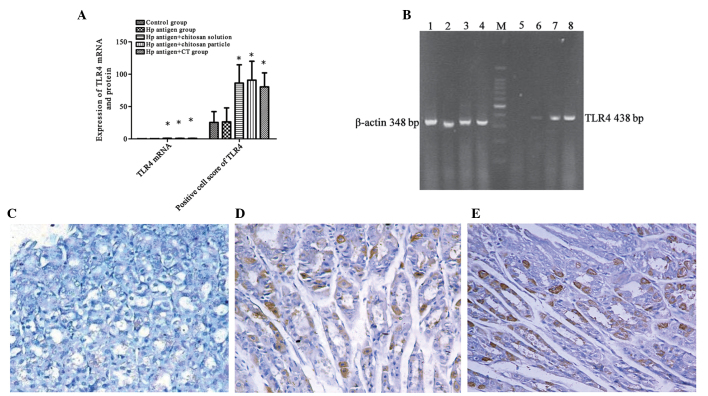
TLR4 mRNA and protein expression levels in the gastric mucosa. (A) ^*^P<0.001 compared with the control group and the Hp antigen group. (B) Lane M, 100-bp DNA ladder; lanes 1–4, β-actin; lanes 5–8, TLR4; lanes 1 and 5, control group; lanes 2 and 6, Hp antigen group; and lanes 3 and 7, Hp antigen + chitosan solution group; lanes 4 and 8, Hp antigen + chitosan particle group. (C–E) TLR4 immunohistochemical staining of the gastric mucosa (magnification, x400). (C) The control group showed few positive cells. (D) The group with chitosan as the adjuvant showed abundant positive cells. (E) The group with CT as the adjuvant showed abundant positive cells. No significant difference was observed between the group with chitosan as the adjuvant and the group with CT as the adjuvant (P>0.05). Hp, *H. pylori*; CT, cholera toxin.

The expression of TLR4 mRNA and the positive cell scores of TLR4 in the gastric mucosa of the vaccine groups were found to be significantly greater than those of the control and the *H. pylori* antigen groups (Fig. 7A–E; P<0.001).

### Expression levels of Foxp3 mRNA and protein in the gastric mucosa

To determine the role of Foxp3 in vaccination therapy following *H. pylori* infection, the expression levels of Foxp3 mRNA and protein were measured in the gastric mucosa by RT-PCR and immunohistochemical staining. In RT-PCR, the amplification primers were: Forward, 5′-GAC GAT ATC GCT GCG CTG-3′; and reverse, 5′-GTA CGA CCA GAG GCA TAC AGG-3′ for β-actin (cDNA length, 348 bp); and forward, 5′-GGC CCT TCT CCA GGA CAG A-3′ and reverse, 5′-GCT GAT CAT GGC TGG GTT GT-3′ for Foxp3 (cDNA length, 218 bp).

**Figure 8:**
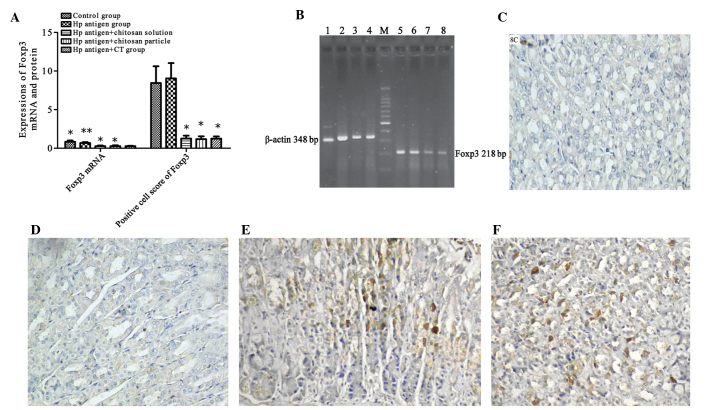
mRNA and protein expression levels of Foxp3 in the gastric mucosa. (A) ^*^P<0.001 vs. the control group and the Hp antigen group; ^**^P<0.05 vs. the control group. (B) Lane M, 100-bp DNA ladder; lanes 1–4: β-actin; lanes 5–8, Foxp3; lanes 1 and 5, control group; lanes 2 and 6, Hp antigen group; lanes 3 and 7, Hp antigen + chitosan solution group; lanes 4 and 8, Hp antigen + chitosan particle group. (C-F) Foxp3 immunohistochemical staining of the gastric mucosa (magnification, x200). (C) The group with chitosan as the adjuvant demonstrated no positive cells. (D) The group with CT as the adjuvant showed no positive cells. (E) The control group revealed sporadic positive cells. (F) The Hp antigen group exhibited abundant positive cells. Hp, *H. pylori*; CT, cholera toxin.

The expression of Foxp3 mRNA and the positive cell score of Foxp3 in the gastric mucosa of the vaccine groups were identified to be significantly less than those of the control and the *H. pylori* antigen groups (Fig. 8A–F; P<0.001, vaccine groups, vs. control and *H. pylori* antigen groups).

## Discussion

To investigate the *H. pylori* therapeutic vaccine with chitosan as the adjuvant, mice were infected with *H. pylori* and vaccinated with an *H. pylori* protein vaccine with chitosan as the adjuvant to delineate the therapeutic effect of the *H. pylori* vaccine and establish the potential mechanism against *H. pylori* infection in comparison with an *H. pylori* vaccine with CT as the adjuvant. It was found that the effect of the *H. pylori* therapeutic vaccine with chitosan as the adjuvant was equivalent to the *H. pylori* therapeutic vaccine with the traditional mucosal adjuvant, CT.

In the present study, the eradication rate of the *H. pylori* vaccine with chitosan as the adjuvant was found to be 58.33%, which was approximately that of the *H. pylori* vaccine with CT as the adjuvant (45.45%), and significantly greater (P<0.005–0.05) than that of the *H. pylori* antigen alone and control groups. These results indicate that chitosan may act as a substitute for CT as a mucosal adjuvant for a *H. pylori* therapeutic vaccine. Furthermore, in mice where the *H. pylori* infection had not been eradicated, the density of *H. pylori* colonization in the gastric mucosa of the *H. pylori*-vaccinated groups with chitosan as the adjuvant was significantly (P<0.05) less than that of the group with CT as the adjuvant, demonstrating that the adjuvant activity of chitosan was stronger than that of CT. Therefore, the present study demonstrated that the degree of chronic gastritis in the groups with chitosan as the adjuvant was significantly less than that of the groups without chitosan as the adjuvant; thus, indicating that the *H. pylori* vaccine with chitosan as the adjuvant reduced *H. pylori*-induced chronic gastritis.

The effect of humoral immunity in *H. pylori* immunotherapy was also investigated in the present study. It was found that the *H. pylori* vaccine with chitosan or CT as the adjuvant elicited anti-*H. pylori* IgG in mice, which may be utilized in immunotherapy. Furthermore, chitosan as the adjuvant in the *H. pylori* vaccine induced a systemic humoral immune response. Similarly, in a study of chitosan co-administered with an influenza virus subunit vaccine, local and serum antibody levels were observed to be markedly enhanced (9). In the present study, it was found that the level of anti-*H. pylori* IgA in saliva and the gastric mucosa in the groups with chitosan as the adjuvant was significantly greater than that of the control group and the groups without any adjuvant (although it was not significantly different from that of the group with CT as the adjuvant). These results indicate that the local secretory IgA antibody may correlate well with the protection function of the *H. pylori* vaccine. Similarly, IgA has previously been presented as critical in immune protection of an *H. pylori* vaccine with CT as the adjuvant (10) and immune protection of an *H. felis* vaccine with LT as the adjuvant (11).

The levels of Th1 cytokines (IFN-γ and IL-12) and Th2 cytokines (IL-10 and IL-4) in the groups administered with a vaccine plus an adjuvant were significantly greater than those of the control group and the groups without an adjuvant. Furthermore, it was found that the *H. pylori* vaccine with chitosan or CT as the adjuvant significantly increased the levels of Th1 and Th2 cytokines in the gastric mucosa of mice, as well as in the sera. The increased anti-*H. pylori* IgG2a and IgG1 levels may induce the mixed immune response of Th1 and Th2. In addition, the ratio of IgG2a to IgG1 in the serum of the groups with chitosan as the adjuvant was found to be less than that of the group with CT as the adjuvant. In addition, the levels of Th2 cytokines, particularly IL-4, in the gastric mucosa of the groups with chitosan as the adjuvant were significantly greater than those of the group with CT as the adjuvant. These results reveal that the *H. pylori* vaccine with chitosan as the adjuvant may recover Th2 immunity that is suppressed by the *H. pylori* infection, as well as regulate the balance of Th1 and Th2 inflammatory cells, thus facilitating clearance of the *H. pylori* infection. A previous study reported that IFN-γ, produced by Th1 cells, induced an increase of the major histocompatibility complex II antigen to recruit inflammatory cells to the gastric mucosa to damage the mucosal tissue, which benefited the clearance of *H. pylori* to a certain degree (12). The *H. pylori* vaccine with Freund's adjuvant was identified to be more effective against the *H. pylori* infection by inducing the mixed reaction of Th1 and Th2 rather than only inducing the reaction of Th2 (13). It was demonstrated in the present study that the effective treatment of *H. pylori* infection by the *H. pylori* vaccine was closely associated with Th1 and Th2 cells. Therefore, chitosan as the adjuvant in the *H. pylori* vaccine may be more effective than CT as the adjuvant for the immunotherapy of *H. pylori* infection, as it is less toxic.

In the present study, following therapeutic vaccination, the expression of TLR4 mRNA in the gastric mucosa and the number of TLR4-positive cells were found to be significantly increased. The low response to *H. pylori* in the gastric epithelial cells may be corrected and the *H. pylori* immune tolerance might be damaged, which may benefit clearance of the *H. pylori* infection. Furthermore in the present study, it was observed that in the vaccination group, the expression of TLR4 mRNA in the gastric mucosae of the mice where the *H. pylori* infection had been eradicated was significantly greater than that of the mice where the *H. pylori* infection had not been eradicated. This indicates that TLR4 may benefit the antibody-mediated *H. pylori* clearance, which is critical in *H. pylori* vaccine immunotherapy. This result is in accordance with a previous study in which TLR4 was determined to be essential for antibody-mediated clearance of bacteria (14).

The effect of regulatory T cells in *H. pylori* immunotherapy was also investigated in the present study. The changes of Foxp3 mRNA and Foxp3-positive cells in the gastric mucosa following vaccination were analyzed and Foxp3 mRNA and the number of Foxp3-positive cells in the gastric mucosa were observed to be significantly reduced. Vaccination reduced the level of CD4^+^CD25^+^Foxp3^+^Treg in the gastric mucosa, relieved the immune response depression and damaged the *H. pylori* immune tolerance, thus benefiting the *H. pylori* infection clearance. In addition, it was found that the expression of Foxp3 mRNA in the gastric mucosa of mice where the *H. pylori* infection had been eradicated was significantly less than that in the mice where the *H. pylori* infection had not been eradicated. Previous studies with an *H. pylori* infection mouse model demonstrated that a certain degree of inflammation is required to reduce the bacterial load in the stomach and that the absence of regulatory T cells is associated with increased gastric inflammation and a decreased bacterial load (15–17), indicating that the enhancement of Treg, induced by *H. pylori* infection, may inhibit the immune response. It was found in the present study that Treg, in CD25-depleted mice, reduced *H. pylori* loads in the *H. pylori*-infected gastric mucosa, increased the numbers of mucosal T cells, B cells and macrophages, and increased the titers of *H. pylori*-specific IgG1 and IgG2a antibodies. These results indicated that the depression of Treg favored *H. pylori* clearance. Therefore, after *H. pylori* vaccine inoculation, the significant depression of CD4^+^CD25^+^Foxp3^+^Treg in the gastric mucosa may participate in the process of *H. pylori* infection clearance.

In conclusion, the *H. pylori* vaccine with chitosan as the adjuvant was found to effectively increase the *H. pylori* elimination rate. Furthermore, the *H. pylori* vaccine with chitosan or CT as the adjuvant induced specific anti-*H. pylori* IgA in the gastric mucosa, and non-specific secretory IgA and specific anti-*H. pylori* IgG in the sera. In addition, the *H. pylori* vaccine with chitosan or CT as the adjuvant promoted Th1 and Th2 cytokines, and decreased the ratio of IgG2a to IgG1. The *H. pylori* vaccine with chitosan as the adjuvant effectively increased the humoral immune response, the Th1 and Th2 cell immune reaction, as well as balancing the Th1 and Th2 response; all of which may contribute to clearance of the *H. pylori* infection. Furthermore, following *H. pylori* vaccination, the TLR4 expression in the mouse gastric epithelial cells increased and the number of CD4^+^CD25^+^Foxp3^+^Treg decreased, which may influence the process of *H. pylori* infection clearance. The effect of the *H. pylori* therapeutic vaccine with chitosan as the adjuvant was equivalent to the *H. pylori* therapeutic vaccine with the traditional mucosal adjuvant, CT. Thus, these findings may promote the use of chitosan as an adjuvant for the *H. pylori* therapeutic vaccine.

## Figures and Tables

**Table I:** *Helicobacter pylori* (Hp) colonization density of a quantitative culture in the gastric mucosa.

Group	n	Median Hp colonization density (colony forming units/g of gastric mucosa)
Control	12	1.4×10^8^
Hp antigen	11	1.8×10^5a^
Hp antigen + chitosan solution	12	0^ab^
Hp antigen + chitosan particle	12	0^ab^
Hp antigen + cholera toxin	11	0^a^

P<0.001.

a P<0.001 compared with the control group;

b P<0.05 compared with the Hp antigen group.
